# Co-Creating a Feasible, Acceptable and Safe Home-Based High-Intensity Interval Training Programme for People with Parkinson’s: The HIIT-Home4Parkinson’s Study

**DOI:** 10.3390/ijerph20095671

**Published:** 2023-04-27

**Authors:** Conrad Harpham, Hilary Gunn, Jonathan Marsden, Luke Connolly

**Affiliations:** School of Health Professions, University of Plymouth, Devon PL6 8BH, UK

**Keywords:** Parkinson’s, HIIT, exercise, co-creation, rehabilitation, neurodegenerative

## Abstract

High-intensity interval training (HIIT) is useful and feasible for some people with Parkinson’s (PwP), although long-term adherence may be problematic. If practical, undertaking HIIT in the home setting could be a way to encourage continued participation. However, no home-based HIIT programme has been developed for this population. Therefore, the objectives of this study were to co-create a feasible, accessible, and safe home-based HIIT programme for PwP, including intervention components and logic model. This supports the longer term aim to assess the practicality and utility of home-based HIIT for PwP. The study included three stages. Firstly, an initial HIIT programme and logic model proposal was developed based on existing evidence. This was refined through an iterative, co-creative process of focus groups, exercise testing and interviews involving end-users and relevant stakeholders. Finally, a draft intervention was produced with further co-creator input. During the iterative process, five focus groups, 10 exercise testing sessions and 10 post exercise interviews were undertaken, involving academic researchers, 6 PwP, one family member and two clinicians. These co-creators developed HIIT-Home4Parkinson’s (HH4P), a 12-week thrice weekly home-based HIIT programme for PwP based on adaptability, individualisation, and remote support. Despite methodological limitations within the development process, the co-created HH4P programme could be feasible, safe, and useful for PwP. A feasibility study should now be undertaken to address remaining uncertainties prior to a full trial.

## 1. Introduction

High-intensity interval training (HIIT) is a specific type of exercise involving alternate bursts of high-intensity exercise and periods of rest or active recovery. HIIT has been frequently cited as an effective, time-efficient exercise modality with which to improve a number of physiological and clinical outcomes in healthy [1] and clinical populations [2,3]. For people with neurodegenerative disorder Parkinson’s (PwP), a recent systematic review [4] concluded that short-term supervised HIIT was a safe and feasible exercise modality with which to improve cardiorespiratory fitness, and possibly motor symptoms and increase brain-derived neurotrophic factor, thought to have important neuroprotective qualities [5]. Additionally, HIIT was concluded to be at least as effective as moderate intensity continuous exercise (MICE) with reduced exercise volume and time commitment. However, due to the lack of studies that included long-term follow-up, along with low adherence in studies of over 12 weeks, the review also concluded that PwP may struggle to adhere to extended HIIT programmes. PwP experience several disease specific and logistical barriers that may limit long-term participation, such as lack of time [6], expense, and difficulty with travel logistics due to motor symptoms [7]. Undertaking HIIT in the home setting could potentially overcome these barriers, and if practical, could be an apposite exercise strategy for PwP. Additionally, given the increasing recognition of the importance of encouraging and facilitating self-management [8], maximising the potential of home-based exercise would seem to be in accordance. While existing evidence suggests that flexibility, balance and strengthening exercises can be undertaken safely and effectively by PwP in the home environment [9], all previous HIIT programmes have been delivered in clinical settings [4]. Given the limitations of remote supervision such as the tendency for participants to perform exercises inaccurately [10], motor symptoms experienced by PwP, and potentially deleterious nature of high-intensity exercise, several uncertainties exist regarding the practicality and utility of home-based HIIT. Therefore, by utilising a co-creative, iterative, mixed-methods development process with key patient and participant (PPI) and clinician involvement, this novel study aimed to co-create a feasible, acceptable and safe home-based HIIT programme with which to assess the practicality and utility of home-based HIIT for PwP.

The objectives were:

1. To develop a home-based HIIT programme which was feasible and achievable for PwP;
2. To develop the intervention components (including clinician and participant materials, guides and recording documents);
3. To develop a first draft of the intervention logic model which would identify the key programme components for process evaluation in future studies.

## 2. Materials and Methods

HIIT-Home4Parkinson’s (HH4P) development was undertaken in three stages. Firstly, an initial proposal for an exercise programme and intervention logic model was developed based on existing literature. Secondly, in line with MRC guidance [11], this proposal was evolved through a three-round iterative, co-creative development process of focus groups and exercise testing with planning, conducting, reflecting, evaluating, and design revision embedded throughout the process. Thirdly, final drafting of the exercise protocol and logic model was undertaken with further input from co-creators.

### 2.1. Stage 1: Exercise Protocol and Logic Model Initial Proposal

As no home-based HIIT programme had yet been developed for PwP, key principles of the initial proposal were informed by relevant existing evidence [4,12] and initial discussions with PwP and clinical experts. Alongside the initial exercise proposal, an intervention logic model was developed based on Medical Research Council (MRC) guidance for the development and evaluation of complex interventions [13]. Constructing a logic model is a key step in the development process, ensuring the articulation of all key elements of the programme. The final logic model is critical to support process evaluation in later feasibility studies, enabling researchers to identify potential design refinements by considering the impact of factors such as implementational process, delivery fidelity, mechanisms of impact and the influence of contextual factors [13].

### 2.2. Stage 2: Evolving the Initial Proposal

The second stage of development was to refine the initial proposal through an iterative, co-creation process. The process of co-creation allows for essential topic specific insight from various user groups and stakeholders, thereby maximising the likelihood of the programme being authentic for the perspective of potential users [11]. Iterative planning is a dynamic element that can be utilised at any level of the co-creation process, considered to be a key principle within the MRC framework within the co-creation of complex health interventions [14]. As a cyclical process, each iteration is used as the starting point for the next repetition with the aim of achieving a specific goal. Three iterative rounds of focus group discussions and practical exercise testing were undertaken, with reflection and evaluation from each round informing subsequent iterations. Initially through an inductive approach, qualitative data collection began the development process through PPI and clinician focus groups. From this point, a deductive approach was utilised with synthesised data informing aspects to be explored within subsequent exercise testing sessions and focus groups.

#### 2.2.1. Co-Creator Recruitment Strategy and Eligibility

The sampling process within co-creation should ensure, “a representative sample of end-users” and, “representation of all necessary expertise from relevant stakeholder groups” [11] (p. 6). Therefore, purposive sampling was utilised to recruit PwP, family members and clinicians over a three-month period. It was originally planned to conduct focus groups with at least six participants per group, in line with previous recommendations to achieve saturation [15,16]. Three focus group panels were proposed, consisting of two PPI panels each involving five PwP, with an additional family member or caregiver per person, and one formed of clinicians—therapists and exercise programme leaders working with PwP. It was planned that five PwP would be recruited to undertake exercise testing. This was considered an appropriate sample with which to extract the required data whilst minimising participant burden. To allow for potential participant drop-out or non-attendance, initial recruitment targets were 20% higher than the required sample [17]. Invitations to participate were initiated through University of Plymouth (UoP) social media accounts and Parkinson’s UK. Co-creators attending focus groups or exercising were required to be of Hoehn and Yahr stages 1–3 (low to moderate disease severity) to ensure discussion group homogeneity [15] and provide similar perspectives regarding HIIT. Additionally, this factor was to maximise participant safety during exercise programme testing, as Harpham et al. [4]. concluded insufficient evidence to suggest that HIIT was a viable exercise option for PwP of greater disease severity. Clinicians were eligible if they were qualified or student physiotherapists, and had experience of leading or being involved in the delivery of exercise classes for PwP. All focus group members were required to have access to the internet. All co-creators received information prior to the study detailing expectations, anticipated time commitment and ethical issues, and completed online consent forms. Co-creators consenting to exercise also completed a bespoke health-screening questionnaire to ensure no pre-existing health conditions were present that could be exacerbated by HIIT.

#### 2.2.2. Focus Group Methodology

Synchronous online focus groups were deemed the most appropriate form of qualitative data collection due to the potential for spontaneous interaction of participants, and the possibility to gauge reaction as a group, as well as individual opinion, leading to the collection of more informative data [18]. Focus group methodology was informed by the work of Morgan et al. [15]. amended to accommodate the use of an online platform. Whilst in-person focus groups could have encouraged greater interaction, it was decided to use an online platform for logistical reasons, therefore minimising participant burden [19], and in consideration of contemporaneous COVID-19 protocols. Separate PPI/clinician groups were undertaken to ensure participant homogeneity [15], and due to PPI advisors expressing a preference for discussing experiences with similar participants. Whilst heterogeneity within focus groups may have allowed for direct quality discussion between co-creators [11] the chosen homogenous format was considered important for uninhibited discussion of potentially sensitive information [15]. Patient opinions in the form of either phenomenological accounts or perceptions of HIIT were considered to be of equal importance for discussion, as both were identified as key contextual factors that could moderate engagement in the exercise programme. To facilitate co-creation, thematic categories from each group were shared with other discussion groups within the iterative round. The chronological order for each round of discussion groups/exercising was; PPI group, clinician group, and lastly exercise testing. Focus groups were 45 min in duration, facilitated by two researchers, one present as an observer to monitor for non-verbal cues and potential signs of fatigue. Initial focus groups included a short introductory Microsoft PowerPoint (PP) presentation (version 2203) with information regarding HH4P, potential outcomes and other relevant findings to date. Focus group question schedules were piloted with other PwP of similar disease stage before main discussions were undertaken.

#### 2.2.3. Focus Group Data Analysis

Focus groups were recorded with Zoom online version 5.10.4 (Zoom Video Communications, CA, USA), and transcribed verbatim. Data were analysed with the use of thematic analysis [20], a technique commonly used to analyse qualitative datasets in sport and exercise research [21]. An inductive semantic approach was utilised for the initial focus group, with a coding framework developed to recognise emerging themes, arranged into major thematic categories and subcategories. The second and third PPI and clinician groups utilised a deductive semantic approach, with pre-defined themes and questions informed by previous discussions and exercise testing. Two researchers (CH and HG or CH and LC) independently undertook data analysis, and identified similar themes and sub-themes. Nvivo (version 11) was utilised to facilitate data analysis.

#### 2.2.4. Exercise Testing Methodology

Supervised laboratory and home-based exercise testing was undertaken to evaluate the feasibility and acceptability of the initial and refined HIIT protocols. As a component in the iterative process, quantitative and observational data from exercise testing along with participant feedback was collected and analysed to inform subsequent rounds. Each participant was asked to undertake three exercise testing sessions, with the initial two laboratory based, and third in participant homes.

#### 2.2.5. Exercise Environment

For laboratory-based exercising, 2 metres^2^ of free space was utilised, along with sufficient height to allow a full vertical stretch. Participants were asked to wear comfortable sports clothing and footwear. As reduced heat tolerance is associated with neurodegenerative conditions, potentially exacerbated by high intensity exercise [22], the exercise environment was thermoneutral (20–25 degrees Celsius) with low relative humidity. A fan was available if required, and water and sustenance was offered after exercising. Participants were asked to adhere to similar protocols as far as possible during home-based exercise testing.

#### 2.2.6. Exercise Session 1

Participant demographic, anthropometric and medical data was initially recorded. Maximal incremental exercise tests (IET’s) were then undertaken using a Lode Corival cardiopulmonary exercise testing cycle ergometer (Lode C.B., Groningen, Netherlands), to determine appropriate individualised HIIT intensity by establishing maximum heart rate (HR_max_) then target heart rate (HR). Maximal oxygen uptake (VO_2max_) was also measured to enable an evaluation of the extent to which HIIT exercises would need to be individualised according to differing levels of fitness. IET protocol included the initial recording of one minute resting data of HR, respiratory exchange ratio (RER) and VO_2_, followed by a ramped adaptation of a protocol previously utilised for PwP [23,24]. Full IET protocol can be seen in Appendix A.

#### 2.2.7. Sessions 1–3: HIIT Programme Testing

In session 1 following the initial IET, participants were given the choice of undertaking exercises from the draft HIIT protocol after an extended recovery period, or completing these in subsequent appointments. HIIT exercises were demonstrated by the PI and rehearsed by the participant to ensure understanding. This was followed by participants selecting exercises to be undertaken, and a 10-min warm-up task. During all HIIT sessions, participants aimed to complete exercises at individualised target intensity. HR was continually recorded using a Polar Beat iPhone application, paired to a Bluetooth equipped Polar H9 HR monitor (Polar, Kempele, Finland), with each session exported as a Microsoft Excel spreadsheet (version 2204) from Polar Flow online software. Additionally, rate of perceived exertion (RPE) [25], set completion, exercise form, adverse effects and other relevant observations were recorded. Immediately after the completion of each HIIT exercise testing session, participants completed a <10-min semi-structured interview with standardised questions to explore perceptions of the undertaken exercises. 

Subsequent exercise sessions involved undertaking the HIIT exercises evolved through focus groups and exercise testing, with the final session conducted in participant homes to explore considerations specific to the home setting. Participants were encouraged to maintain normal physical activity levels and dietary habits for the duration of the three rounds of exercise testing, with any changes in medication/medical circumstance reported to researchers.

#### 2.2.8. Exercise Testing Data Analysis

Following each HIIT session, the mean HR and RPE achieved by each participant per exercise set, and overall mean HR/RPE per session were calculated. Additionally, mean HR and RPE were calculated per exercise to establish the intensity of each differing modality. Tabulation of other feasibility factors such as set completion, exercise form and adverse effects was undertaken. After the completion of the three iterative rounds, the overall mean (standard deviation [SD]) HR, and mean RPE were calculated per participant and exercise. Post-exercise interview data was recorded with a laptop voice recorder application, transcribed verbatim, and analysed with thematic analysis techniques [21].

### 2.3. Stage 3: Final Drafting

Following consideration of qualitative and quantitative data synthesised throughout the iterative co-creation process, a draft of the co-created exercise programme and logic model was produced. This was sent to all co-creators for concluding thoughts, to suggest potential modifications, and to offer reflections of the overall development process. Upon this feedback, final versions were then completed.

## 3. Results

### 3.1. Stage 1—The Initial Proposal

#### 3.1.1. Key Principles

With the aim of developing a programme suitable for home use, the initial iteration of the HH4P protocol and logic model was based on several key principles. The HIIT protocol was proposed to be undertaken thrice weekly over a 12-week period [4]. Thiswould consist of a variety of adaptable exercises to allow for specific preferences and health-related and logistical needs of the individual [26]. The protocol would include four sets of exercises focusing on differing muscle groups, interspersed with periods of rest [4]. Several types of exercises would be considered, including calisthenic, and adaptations of resistance exercises for PwP [12]. Due to the home-based nature of the programme, only the use of minimal equipment would be considered. All exercise protocols would need to be suitable for PwP of mild-to-moderate disease severity (Hoehn and Yahr 1–3) [4], practical within the home environment, and performed at an intensity of ≥75% individualised HR_max_ [27].

The HH4P programme was to be based on remote supervision, utilising appropriate physical and online exercise resources and programme delivery support.

#### 3.1.2. Key Uncertainties

Whilst these principles formed the foundation of the initial proposal, several uncertainties needed to be addressed during the co-creation stage. Firstly, the specific exercises, work: rest ratio and overall exercise duration that were safe, achievable and acceptable needed to be established. Additionally, it was unknown if or how the required HIIT intensity of 75% HR_max_ could be achieved in the home environment without the use of specialist equipment. Whilst exercise variety and adaptability were key principles of the initial proposal, how to adapt the programme, and the extent to which it would need to be individualised according to disease stage or aerobic fitness were unknown. Similarly, to what extent participants would be able to undertake the programme in the “off” phase, when symptoms were more severe was a key uncertainty. Also, contextual factors such as lack of motivation, confidence and family support were initially identified as aspects that could moderate engagement in home-based exercise. Elucidation of the specific strategies and resources to manage these factors and maximise participation in the programme was an important consideration for stage two., Furthermore, outcome measures that were deemed acceptable and feasible by co-creators needed to be established. The initial logic model proposal can be seen within Supplementary Material.

### 3.2. Stage 2—Evolving the Initial Proposal

#### 3.2.1. Co-Creator Recruitment

Out of six PwP who volunteered as a co-creator, five were recruited for PPI focus groups along with one additional family member. Four of these PwP were also recruited for exercise testing, with one additional participant recruited for exercising only, as they did not wish to participate in focus groups. One PPI focus group participant did not consent to exercise sessions due to logistical reasons. All PwP had an interest in exercise, and undertook at least 140 min of moderate-to-vigorous physical activity per week.

Four co-creators were recruited for clinician focus groups; these included three qualified and one student physiotherapist, all of whom were involved in leading or assisting with the delivery of exercise classes for PwP.

#### 3.2.2. Attendance (Figure 1)

The initial PPI focus group and exercise testing were attended by six and five people respectively. All five exercise co-creators undertook IET’s, and target HR was calculated accordingly. One (participant 5) underwent a faulty VO_2max_ test, therefore target intensity was based on age-predicted formulas (220 minus age). This participant withdrew from the study after round one due to an unrelated knee injury. In the following rounds, the number of PPI attendees reduced to four in both focus groups, and three and two for exercise testing in the second and third round respectively due to participant unavailability. Throughout the process, two exercise co-creators reduced in bodyweight, (participant 1, −4.8 kg, participant 2, −5.0 kg), attributing the change to increased levels of habitual physical activity and dietary changes respectively. Also, participant 1 reported a change in dopaminergic medication in round three.

Of the clinicians, two attended two focus groups (one qualified therapist, one student), two did not attend either, and the final group was cancelled due to unavailability. Co-creator characteristics and IET results can be seen in Table 1.

**Figure 1:**
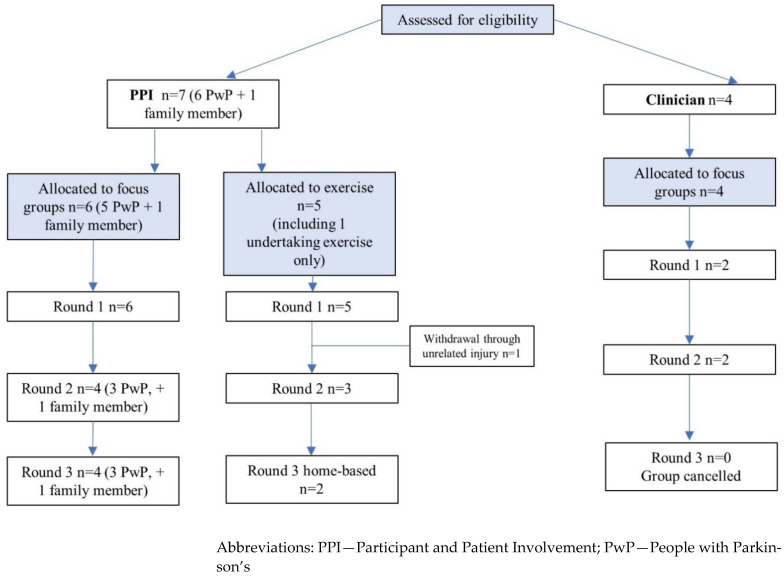
Schematic of co-creator recruitment and attendance.

#### 3.2.3. HIIT Testing Results (Table 2)

Exercise co-creators undertook a total of 40 sets (120 bouts), of various HIIT exercises across the iterative phases, completing 39 in full. Participants achieved mean target intensity in 86% of sets, with an overall mean (SD) intensity of 80.8% (±7.2) HR_max_, and RPE of 14.7 (hard). Adverse effects included mild shoulder pain during lateral shoulder raises (omitted from the programme after round 1), shoulder pain during star jumps following shoulder press, and knee ache during squatting exercises. There were no major adverse effects or events. All individual exercises stimulated an overall mean intensity of >75% HR_max_ with mean RPE ranging from 12.5 (moderate) to 16.0 (hard). No limitations were observed during round three, home-based exercise testing, with exercise intensity uniformly >84% HR_max_ and equipment utilised appropriately. With the exception of one participant who experienced no on/off differentiation, participants undertook exercises in the “on” phase, and all completed interviews following each session.

**Table 2:** Cumulated HIIT testing results per participant.

Participant	HIIT Sets Undertaken/Completed	% of Sets Achieving Mean Target Intensity	Mean (SD) % of HR_max_	Mean RPE	Adverse Effects
1	8/8	100	83.6 (±4.3)	13.4 (Somewhat hard)	Mild shoulder pain
2	7/8	88	80.6 (±5.0)	14.5 (Hard)	Mild shoulder pain
3	8/8	50	71.9 (±6.5)	13.4 (Somewhat hard)	None
4	12/12	92	83.0 (±7.1)	14.9 (Hard)	Mild knee pain/calf cramp
5	4/4	100	86.3 (±1.6)	17.5 (Very hard)	None
Overall	39/40	86	80.8 (±7.2)	14.7 (Hard)	-

#### 3.2.4. Qualitative Data

In total, five focus groups and 10 post exercise interviews were undertaken. Several themes were identified following thematic analysis. These included; concerns and opportunities regarding exercise achievability and differentiation, remote model of support, home-based limitations and outcome measures. Table 3 presents a round by round summary of identified thematic categories and sub-categories.

Full exercise testing results per round, graphical examples of participant HR data, cumulated results per exercise, and examples of focus group transcript datacan be seen in Supplementary Material.

### 3.3. Stage 3: Final Drafting

In the final phase, a draft exercise protocol and logic model were sent to all co-creators. Four (two PwP and two clinicians) gave further feedback, all affirming the suitability of the programme. One co-creator commented that the programme constituted an accurate representation of data obtained throughout the process. No further amendments were made following evaluation of this feedback.

### 3.4. The Final Co-Created HH4P Programme

Following the three stage iterative process, co-creators had developed the HH4P programme, components and logic model based on five key factors to potentially maximise safety, feasibility and adherence. These were; individualised HIIT protocol (including factors such as exercise type, intensity, sequence), minimal exercise resource requirements, adaptability, appropriate programme delivery support, and acceptable outcome measures.

#### 3.4.1. Individualised HIIT Protocol (Table 4)

Throughout the development process various HIIT exercise options were either deemed appropriate for the programme, or omitted due to unsuitability (considering factors such as safety, intensity and variation). All co-creators agreed that participants be given a “menu” of exercise/sequence choices to accommodate differing abilities and preferences. The HIIT protocol will therefore consist of four exercise sets utilising differing muscle groups; set one consisting of whole-body exercises, set two upper-body, set three a variation of whole-body exercises, and set four lower-body, each with a choice of exercises. Participants will choose one exercise per set. HIIT exercises will be a combination of adapted bodyweight HIIT programmes for healthy populations [28,29], calisthenic exercises suggested in focus groups, and adaptations of gym-based HIIT programmes for PwP [12] utilising minimal dumbbells (0.5–2 kg) and resistance bands of differing tensions. HIIT timings will consist of three forty-five second bouts of high-intensity exercise per set, with each bout interspersed with a 15 second period of rest. A longer rest/active recovery period of two minutes will be undertaken between each set. A 10-min warm up session will be completed before the exercise, and a cool down period will follow at the end of the session. In total, the exercise session will last for 32 min, with nine minutes spent undertaking high-intensity exercise. HIIT work phase initial target intensity will be proposed as ≥75% of individualised maximum heart rate [27], and two-minute active recovery periods targeted at 50% HR_max_ as per American College of Sports Medicine (ACSM) recommendations [30]. Participants will be advised to follow the recommended group order, but to discuss amendments with researchers if required. Target intensity will be individualised by participants undertaking IET’s to establish target intensity (75% HR_max_) before the programme. As there was no observable difference between exercise co-creators in the ability to complete HIIT exercises at the relevant intensity, despite differing VO_2max_ scores, disease severity and amounts of habitual physical activity, it was deemed that 75% HR_max_ was an inclusive initial target intensity for the programme. Also, HIIT testing sessions were predominantly completed with participants in the “on” phase—therefore, participants will be advised to complete the HH4P programme while “on”. The HIIT protocol will be undertaken thrice weekly for 12 weeks as initially proposed on non-consecutive days to allow suitable recovery [31].

**Table 4:** The final HH4P co-created HIIT protocol.

Phase	Protocol/Bouts	Intensity	Accumulated Time (min/s)
Warm up	10 min: stretching/fast marching on the spot	50–60% HR_max_	10.0
HIIT set 1	Group 1: Whole body (45 s work/15 s rest) × 3	≥75% HR_max_	12.45
2-min rest/active recovery	Rest/slow walking on the spot	50% HR_max_	14.45
HIIT set 2	Group 2: Upper body (45 s work/15 s rest) × 3	≥75% HR_max_	17.30
2-min rest/active recovery	Rest/slow walking on the spot	50% HR_max_	19.30
HIIT set 3	Group 3: Whole body (45 s work/15 s rest) × 3	≥75% HR_max_	22.15
2-min rest/active recovery	Rest/slow walking on the spot	50% HR_max_	24.15
HIIT set 4	Group 4: Lower body (45 s work/15 s rest) ×3	≥75% HR_max_	27.0
Cool down	5 min walking on the spot (voluntary speed)/stretching	Back to resting	32.0
Menu of HIIT exercises			
Group 1: Whole body Running on the spot, or Skipping (without rope), or Star jumps	Group 2: Upper body Boxing (Crosses, jabs, hooks and uppercuts), or Over-head shoulder press with choice of hand-held weights, or Front arm-raise with choice of resistance bands	Group 3: Whole body (Select different exercise from choice 1 if possible) Running on the spot, or Skipping (without rope), or Star jumps	Group 4: Lower body (Performed with balance aids/supports as required) Body-weight squats, or Chair sit to standing position, or Forward lunges

Total time per session: 32 min (Including 10 min warm-up, 5 min warm down). Total time spent in high-intensity exercise: 9 min. Programme duration/frequency; 12 weeks 3 x per week, non-consecutive days. Participants can adapt the sequence as necessary according to preference/abilities. Abbreviations: HIIT—High intensity interval training; HR_max_—maximum heart rate; BPM—Beats per minute; min/s—Minutes/seconds.

#### 3.4.2. HIIT Exercise Resources

Co-creators recommended that exercises be accompanied by rhythmic auditory cueing (RAC). RAC is an audial stimulus such as music or metronome, that can accompany exercise with the aim of synchronizing motor execution and rhythms, thereby maintaining cadence, through the use of continuous isochronous beats [32,33]. Co-creators agreed that the addition of RAC in the form of musical accompaniment was useful for maintaining exercise cadence and movement amplitude, and therefore intensity, along with increasing enjoyment. Following piloting, RAC tempo of 130–135 BPM was found to be apposite for synchronisation to the selected HIIT exercises. This is congruent with previous research suggesting tempi within this range to be suitable for maintaining motor task synchronicity during high-intensity exercise [34]. Additionally, pre-recorded HIIT timing instructions and encouragement were deemed to be of importance. However, co-creators raised the concern that music may not be to the taste of the individual. Therefore, a stylistic range of original, musical RAC will be available with instructions and encouragement, as either CD or downloadable mp3 files to maximise accessibility. Co-creators also agreed that balance aids as available in the home setting (chairs on a flat surface), visual exercise prompts as a reminder of chosen exercise sequence, and copies of a user friendly exercise menu were acceptable and useful. HH4P will also be delivered with minimal exercise equipment as previously described. Resources will also include a Polar HR monitor and chest strap, paired to the Polar “Beat” application to be downloaded on participant smartphones. Exercise piloting indicated the initially proposed two metres^2^ of space to be appropriate.

#### 3.4.3. Adaptations

Co-creators observed the potential of several exercises to be adapted, to individualise according to ability. Therefore, exercises will be adapted through;

- The potential to double (or halve) exercise frequency, thereby modifying the intensity.
- The potential to “bounce” (inclusion of lower body) during shoulder exercises and boxing, thereby increasing the intensity.
- Utilising home-based balance aids for support/leverage to maximise safety and achievability during lower-body exercises.
- The potential to add an extra coordinative aspect, thereby increasing the difficulty level (and potentially providing additional benefit to static and dynamic balance) [35]. For example, boxing exercises could also involve an element of foot rotation if required.
- To account for physiological adaptation to the programme, co-creators recommended that exercise target intensity should be titrated to maintain a suitable relative intensity, depending on progression. This will be addressed in fortnightly check-ins, and target exercise intensity titrated up (or down) accordingly. Increased intensity will be achieved by increasing movement amplitude and/or frequency, or through exercise adaptations (for example “bouncing” during boxing).

#### 3.4.4. Programme Delivery Support

A key consideration for co-creators was the home-based nature of the intervention, and associated lack of supervision potentially leading to safety and motivational issues. Consequently, the following aspects were included in the model of support;

- Baseline assessment visit to UoP will give the opportunity for uncertainties to be addressed in person.
- Pre-intervention home visit will include exercise technique and adaptations training, equipment provision and instruction, remote support instruction, partner liaison and exercise location preparation.
- Fortnightly online check-ins will provide motivation and assess for potential programme adaptations.
- Fortnightly online check-ins will monitor for adverse effects/events.
- Exercise reminder texts will be sent to participants.
- Monthly online group sessions will provide opportunities for social interaction.
- A range of internet-based and physical support resource will be available, which will include; exercise demonstration instructional video performed by a PwP, audio RAC accompaniments, contact information for live feedback, diaries and recording sheets, HIIT information sheets, loaned HR monitor and accelerometer along with equipment care and instruction leaflets.
- User-friendly diaries to record adherence and completion, substantiated by online HR data.
- Researcher contact details for immediate feedback.

#### 3.4.5. Outcome Measures

Along with practicality outcomes such as exercise completion, adherence, adverse events and achieved intensity, the following were selected to represent a range of physiological, mechanistic, clinical and patient-centric assessments deemed by co-creators to be valid, feasible and important. These were; serum levels of endogenous protein brain-derived neurotrophic factor (BDNF), VO_2max_ (using the piloted protocol), Movement Disorder Society Unified Parkinson’s Disease Rating Scale part III (UPDRS III), 30 Second Sit-To-Stand Test (30 s STS), and the Oxford Participation Activities Questionnaire (OxPAQ) acute. Co-creators also agreed that habitual physical activity measurement with loaned accelerometers for one week, both at the start of the programme and at week seven, was acceptable and important to nullify this potential confounding variable. 

Appendix B shows the final co-created logic model, highlighting key aspects of the HH4P programme.

## 4. Discussion

This study aimed to co-create a safe, feasible and acceptable home-based HIIT programme for PwP. To our knowledge, this is the first study to utilise a co-creative, iterative development model with focus groups and integrated exercise piloting to develop an exercise programme for PwP. The HH4P HIIT protocol, logic model, outcomes and study resources were successfully developed through synthesis of existing evidence and qualitative and quantitative data collected during the study. High rates of exercise session completion, achieved exercise intensity, lack of adverse effects and events, absence of obvious home-based limitations, and qualitative participant feedback has maximised the likelihood of the programme being successfully utilised. The apparent feasibility of HIIT exercises for PwP of mild-to-moderate disease severity is congruent with previous research [4,12] that concluded HIIT, albeit when supervised, was achievable for this sub-section of the population. However, as recommended by Gallo [26], HIIT safety and intensity within this study was facilitated through the continual adaptation of protocol to allow for individual abilities and preferences. As observed by co-creators, any minor adverse effects during the development stage were attributed to inappropriate individualisation of exercise choice and sequencing. Appropriate exercise intensity was mostly achieved throughout the process—only one exercise co-creator did not achieve overall mean target intensity (71.9% HR_max_). However, this participant expressed uncertainty as to the amount of required effort during one session, which could have influenced results. The inclusion of a musical RAC accompaniment was also deemed important by co-creators, and is supported by recent evidence. RAC is theorised to ameliorate motor symptoms in PwP by externally bypassing Parkinson’s related rhythmic deficit internal to the basal ganglia [36], and has been evinced to provide additional benefit to balance, gait and mobility [37]. The use of music, as opposed to atonal metronome beats as an auditory accompaniment is hypothesised to offer further benefit to exercise performance through anxiety control [38], and fatigue disassociation [39].

### 4.1. Programme Delivery Support

Lacroix et al. [10]. discuss several potential limitations of home-based exercise for older adults, citing factors such as reduced intensity and safety issues as a consequence of improper exercise execution through lack of supervision. Congruently, and in agreement with Paul et al. [7]. co-creators within this study identified appropriate supervision as a key aspect of programme delivery. Therefore, careful consideration of the most appropriate remote model of support was key when developing the HH4P programme. For example, as utilised in a recent home-based feasibility study for people with progressive multiple sclerosis [40], the potential opportunities afforded by an initial home visit became apparent throughout the process. A proposed inclusion of this visit, supervised participant exercise training has been suggested as an important component of previous home exercise programmes for PwP that were completed with high adherence rates (>85%) [41,42]. Although these studies were of lower intensity exercise modalities, this aspect was considered to be a suitable component for HH4P. Similarly, online resource packages and regular remote researcher communication have been commonly utilised within home-based exercise training programmes for older adults and PwP [9,43], and can enhance adherence and exercise execution [43]. However, the inclusion of participant diaries to record adherence, completion and other data could be debated. This form of self-reporting has been cited as a limitation within home-based exercise programmes for PwP, due to potential tremor and putative forms of bias such as selective recall and social desirability potentially influencing results [9]. Nonetheless, whilst the intended use of diaries within the HH4P programme is intended to provide an element of data, the addition of objective, online HR monitoring was considered key by co-creators to substantiate exercise completion, adherence and achieved intensity.

### 4.2. Outcome Measures

The inclusion of BDNF reflects the importance co-creators (particularly PwP) attributed to mechanistic outcomes that could contribute to slowing of disease progression—the potential neuroprotective qualities of BDNF have been well documented [5]. Additionally, BDNF has been evinced as sensitive to change in non-home based HIIT programmes [44,45], and is therefore a key indicator of the comparative utility of HIIT based in the home setting. Similarly, VO_2max_ has also been increased by previous HIIT programmes [4], and identified as important and acceptable by co-creators, as increased VO_2max_ is associated with a reduced risk of comorbid conditions such as cardiovascular disease [46,47]. Whilst these physiological variables represent important health-related outcomes for PwP, clinical outcomes such as the 30 s STS, MDS-UPDRS III and OxPAQ were included by co-creators to represent important patient-centric factors that could be more pertinent regarding continued participation. Furthermore, as with BDNF and VO_2max_, the UPDRS III has been improved in previous non-home-based HIIT programmes [48].

### 4.3. Limitations of the HH4P Development Process

Whilst the iterative co-creation process guided by MRC recommendations allowed critical PPI and clinician input along with end user exercise piloting, a number of limitations within the development process, and therefore uncertainties remain. Firstly, whilst exercise piloting suggested the feasibility of the HIIT protocol, the practicality and safety of the protocol undertaken thrice-weekly over a continuous 12-week period remains unknown. Also, the number of participants contributing to focus groups, particularly clinician discussions was less than expected. Due to limitations within the recruitment process such as the amount of participant identification centres, the anticipated sample size was not achieved, and the third clinician group was cancelled, potentially limiting valuable insight into specific home-based considerations. Health professionals having limited opportunities for participation has been suggested as a potential limitation of the complex health intervention co-creation process [49] and was found to be a germane factor within this study. However, whilst it was unlikely that data saturation was reached on a micro level, due to the nature of the iterative methodology, it was possible that saturation was reached upon completion of the overall process. Additionally, whilst the inclusion of student clinicians within focus groups potentially allowed for a more diverse range of perspectives, the lack of clinical experience could have reduced the validity of focus group outputs. 

Participant attrition has been cited as a potential limitation of iterative models of data collection [50]. Congruently, HH4P development involved only two co-creators who undertook home-based HIIT sessions due to increased unavailability by the third round. The development of HH4P could have benefitted from the introduction of home-based testing earlier in the process, thereby increasing participation. Both co-creators undertook exercise in similar home environments, therefore the opportunity to assess the impact of factors such as differing amounts of available space or differing floor types was not afforded, restricting the generalisability of results. The lack of home-based testing also raises uncertainties regarding the suitability of resources, although no limitations were observed within the two sessions undertaken. Participation bias could also have influenced results [51]—all participants had an interest in exercise, and undertook moderate intensity exercise on a daily basis. The development process could have benefited from the inclusion of sedentary participants, to enhance to the generalisability of results to the wider Parkinson’s population. Therefore, whilst the included participants demonstrated a range of disease progression and fitness levels, the suitability of the programme for PwP of mild to moderate severity with more advanced symptoms or further reduced aerobic fitness remains to be established. Also, whilst the inclusion of a pre-focus group PP presentation facilitated understanding, the information provided regarding the evinced benefits of HIIT may have biased discussion, including outcome measures PwP deemed to be of importance. For example, the preference of BDNF as a potential outcome measure may not have been considered if not previously identified within the presentation. Finally, whilst stage three of development allowed for further co-creator feedback, the addition of a focus group upon study completion may have allowed for more informative data regarding perceptions of the iterative co-creation process.

### 4.4. Implications for Future Studies and HH4P

Future studies undertaking exercise intervention development with the iterative, co-creation process should ensure that appropriate clinician recruitment procedures, along with a flexible study timescale are in place to allow adequate participation. Also, potential attrition should be considered when sequencing components of programme development. Furthermore, the inclusion of sedentary participants, and those of differing diseases stages within the parameters of eligibility criteria would increase the generalisability of results. 

For HH4P, when considering both positive and limiting factors within programme development, it is clear despite encouraging results, that uncertainties regarding home-based HIIT for PwP remain. Therefore, it is now critical to undertake a 12-week feasibility study in line with CONSORT recommendations [52,53] prior to a full trial, to assess the delivery of the HH4P programme and proposed evaluation methods.

## 5. Conclusions

The HH4P programme was successfully co-created through synthesis of existing evidence and data collected within an iterative development process. HH4P could be feasible, acceptable and safe for PwP, and may constitute an accessible way to incorporate high-intensity exercise into daily routine, and possibly facilitate extended adherence. However, a feasibility study should now be undertaken to address remaining uncertainties, and to inform methodological considerations for the implementation of a full trial.

## Figures and Tables

**Table 1:** Exercise co-creator baseline characteristics, incremental exercise testing results and focus group participant characteristics.

Exercise co-Creator Baseline Characteristics									Maximal Exercise Testing Results					
Participant	Age (y)	Sex	Height/Weight (cm/kg)	Estimated Weekly MVPA (min)	H & Y Stage	Time Since Diagnosis (y)	Medication/on or off	Age Adjusted HR_max_ (BPM)	Time/Reason for Termination	VO_2max_ (ml/kg/min)	HR_max_ (BPM)	RPE on Termination	RER on Termination	HIIT Target Heart Rate (BPM)
1	63	M	176.5/89.8	180	3	3	Sinemet/Ropinirole: No on/off periods	157	16.56/Volitional exhaustion	35	136	18	1.19	103
2	62	F	164.5/67.8	480	2	2	Madopar: On	158	9.45/Volitional exhaustion	22	144	19	1.19	108
3	49	M	176/77.7	350	3	6	Ropinirole/Madopar/Opicapone/On	171	14.50/Volitional exhaustion/HR & VO_2_ plateaux	36	166	18	1.25	125
4	61	M	177/75.2	210	2	6	Madopar/Rasigiline/On	159	13.06/HR and VO_2_ plateaux	31	163	18	1.25	122
5	64	M	177/80.6	140	3	20	Subcutaneous levodopa/Metazopine/On	156	4.00/Equipment malfunction	- *	-	-	-	117 **
**Focus group co-creator characteristics**														
Group	Participants													
PPI:	Exercise co-creators 1, 2, 3, 4 + 1 PwP (3.5 years diagnosed, H & Y 2, teacher of remedial exercise classes, regular weekly MVPA) + 1 family member (spouse of participant 2)													
Clinician:	1 qualified, registered physiotherapist, with current and over 10 years’ experience of leading exercise classes for PwP including high-intensity programmes + 1 student physiotherapist with current and three years’ experience of assisting with delivering exercise classes for PwP													

* Participant 5 incremental exercise test terminated prematurely due to equipment malfunction; ** Based on age adjusted formula 220-age. Abbreviations: y—years; cm—centimetres; M—male; F—female; kg—kilogram; MVPA—moderate to vigorous physical activity; min—minutes; H & Y—Hoehn and Yahr; HR_max_—maximum heart rate; BPM—beats per minute; VO_2_—Oxygen uptake; VO_2max_—maximal oxygen uptake; ml/kg/min—millilitres per kilogram per minute; RPE—rate of perceived exertion; RER—respiratory exchange ratio; HIIT—high-intensity interval training.

**Table 3:** Overview of key themes identified from focus groups and post exercise interviews.

Round	Event	Attending Co-Creators	Main Thematic Categories (in Bold Type) and Sub-Categories
1	PPI focus group	2 academic researchers 5 PwP, 1 family member	HIIT exercise concerns: Time restraints/Individual capacity/On-off periods/Space restraints/Coordination Programme delivery concerns: Supervision/Injury/Motivation/No social element/Screening HIIT exercise opportunities/motivators: Exercise suggestions/Initial protocol suitable intervals/Potential adaptations/Equipment suggestions Programme delivery opportunities/motivators: Home-based convenience/Remote support suggestions/Evidence of change as a motivator/Initial protocol suitable duration and frequency
	Clinician focus group	2 academic researchers 1 qualified clinician 1 student clinician	HIIT exercise barriers and considerations: Parkinson’s severity differentiation/Reduced movement amplitude Programme delivery concerns: Remote support/Licensing/Motivation/Outcome measures (PDQ-39 unsuitable)/Physio costs HIIT exercise opportunities and facilitators: Rhythmic cueing/Differentiation possibilities/Exercise suggestions: boxing variations Programme delivery opportunities and motivators: Remote support suggestions/Outcome measures/Motivation/Training/Social motivation
	Post exercise interviews	5 PwP (Undertaken separately with 1 academic researcher)	Exercises and timings: Achievable/Challenging but enjoyable/Acceptable duration/Visible heart rate monitor useful/Intensity effort dependent Pre-programme training: No fitness training required, but may be for some PwP/Technique instruction important Safety: Balance issues during squats for those of Hoehn and Yahr 3/Support required for lower-body exercises RAC accompaniment: Correct tempo/Helps to maintain cadence/Enhances enjoyment/Not personal style preference/Differing styles required Other thoughts: Online group sessions may increase motivation
2	PPI focus group	2 academic researchers 3 PwP 1 family member	Outcome measures—acceptability and deemed importance: Brain-derived neurotrophic factor acceptable and important/VO_2max_ acceptable procedure/Physical activity, 2 weeks accelerometery acceptable 30 s sit to stand acceptable but not suitable as fortnightly “motivator”/Rate of perceived exertion concern with judgement/Unified Parkinson’s Disease Rating Scale part III important and acceptable/Adherence, completion, adverse effects and events self-report diary acceptable/Ox-PAQ acute important and acceptable Specific points regarding the HIIT protocol: Challenging and enjoyable/Alternative exercises—core exercises, leg raises/Accompaniment acceptable, but requires variation/Pre-recorded verbal encouragement acceptable/Visible heart rate monitor acceptable and useful/Squats & sit to stand require support/Fixture and fittings damage potential
	Clinician focus group	2 academic researchers 1 qualified clinician 1 student clinician	Outcome measures: Range of outcomes to allow for individualised motivation/Additional motivational check-ins resource intensive/Goal Attainment Scale achievable procedure but problematic Programme delivery: Fortnightly motivational check- in/Proposed programme has multiple options/Ensure choice of options to avoid overloading/Use of Smart phone application Specific points regarding the HIIT protocol: Squats and sit-to-stand balance safety and “lunge” alternative/Core exercises stimulate inadequate intensity/Shoulder exercises overly similar/Alternative exercises to facilitate clinical prescription/Modify sequence to include extra “whole body” set
	Post exercise interviews	3 PwP (Undertaken separately with 1 academic researcher)	Revised exercises and sequence: Suitable duration and timings/As challenging as previous round/Felt “aerobic”/Incorrect sequencing increases risks of injury Lower-body exercise safety: Balance supports increased confidence and achievability/Overbalancing forward during squats, unsuitable for some PwP Amended RAC: Verbal encouragement acceptable and useful/Correct tempo/“Dance” style better than “Blues” for maintaining cadence Exercise sequence prompting cards: Useful/Larger versions required Other thoughts: None
3	PPI focus group	2 academic researchers 3 PwP 1 family member	Considerations of the home setting—increase engagement: Opportunities within initial home visit: prepare environment and engagement with partner/Partner support/Potential to exercise outside Reduce engagement: Exercise environment: Surface, available space, potential damage/Lack of partner support/Motivation/Footwear
	Clinician focus group	Cancelled due to unavailability	N/A
	Post exercise interviews (Home)	2 PwP (Undertaken separately with 1 academic researcher)	Suitability of the home-environment: Positives: Exercises appropriate for the home/User-friendly resources/Required space acceptable/Enjoyable/Good exercise adaptability Negatives: Potential over-reliance on internet access/Importance of initial visit to prepare environment/Alternative format for exercise sequence prompting cards required

Abbreviations: PwP—People with Parkinson’s; PPI—Public patient involvement; RAC—Rhythmic auditory cueing; PDQ-39—Parkinson’s disease questionnaire 39; OxPAQ—Oxford participation activities questionnaire; N/A—Non-applicable.

## Data Availability

The data presented in this study are available in Supplementary Material, and by request to the corresponding author.

## Supplementary Materials

The following supporting information can be downloaded at: https://www.mdpi.com/article/10.3390/ijerph20095671/s1, Figure S1: Initial logic model proposal; Tables S1–S7: Full HIIT testing results; Tables S8–S16: Examples of focus group transcript data; Figures S2–S6: Graphical examples of participant heart rate data.

## Abbreviations

PA—physical activity; HIIT—high-intensity interval training; PwP—people with Parkinson’s; HRmax—maximum heart rate; UoP—University of Plymouth; CI—chief instructor; RPE—rate of perceived exertion; BDNF—brain-derived neurotrophic factor; UPDRS; Unified Parkinson’s Disease Rating Scale; OxPAQ—Oxford Participation Activities Questionnaire; 30 STS—30-second SIT-to-Stand Test; VO2max—maximal oxygen uptake.

## Appendix A. Maximal IET Procedure

IET’s were undertaken using a Lode Corival cardiopulmonary exercise testing (CPET) cycle ergometer (Lode C.B., Groningen, The Netherlands). Oxygen consumption was measured with a face mask connected to a Cortex 3B-R3 (Cortex Biophysik, Leipzig, Germany), and HR measured with a Bluetooth equipped Polar H9 HR monitor. Cortex calibration was undertaken prior to exercise testing, participant anthropometric data entered, and a pre-programmed study protocol was selected. IET protocol included the initial recording of one minute resting data of HR, respiratory exchange ratio (RER) and VO_2_, followed by a ramped adaptation of a protocol previously utilised for PwP [23,24]; IET’s began at 25 watts (w) resistance, with 25 w ramped increments over a two-minute period. Participants were instructed to maintain a pedalling rate of 50 revolutions per minute (RPM), with an on-screen revolutions counter utilised to facilitate the correct cadence. HR, RER and VO_2_ were recorded every second, and HR was also manually recorded every two minutes. RPE was also recorded every two minutes. Tests aimed to reach termination within eight to twelve minutes of commencement, although could be extended past this time. Tests were terminated when participants achieved maximal exercise capacity (oxygen uptake/HR remained constant despite increased workload), on volitional exhaustion, or if participants consistently failed to maintain the correct pedalling rate. A maximum RER of approximately 1.1 to 1.2 was utilised as a supporting criterion of true maximal oxygen uptake [54]. IET’s were accompanied by two researchers, and standardised verbal encouragement was offered throughout [55]. IET results were exported to a Microsoft Excel (version 2204) spreadsheet. VO_2max_ and HR_max_ were determined using a 20-s period average value, to nullify anomalous variations [56]. Individualised HIIT minimum target intensity was then calculated as 75% of achieved HR_max_. Researchers closely monitored IET participants, observing for adverse effects. The full IET protocol was piloted by three exercise specialists prior to being undertaken by co-creators.
