# Supplementary material for: Co-Creating a Feasible, Acceptable and Safe Home-Based High-Intensity Interval Training Programme for People with Parkinson’s: The HIIT-Home4Parkinson’s Study
Source: Int J Environ Res Public Health. 2023 Apr 27;20(9):5671. doi: 10.3390/ijerph20095671 (PMC10178442; doi:10.3390/ijerph20095671)

Supplementary material

Figure S1: Initial logic model proposal

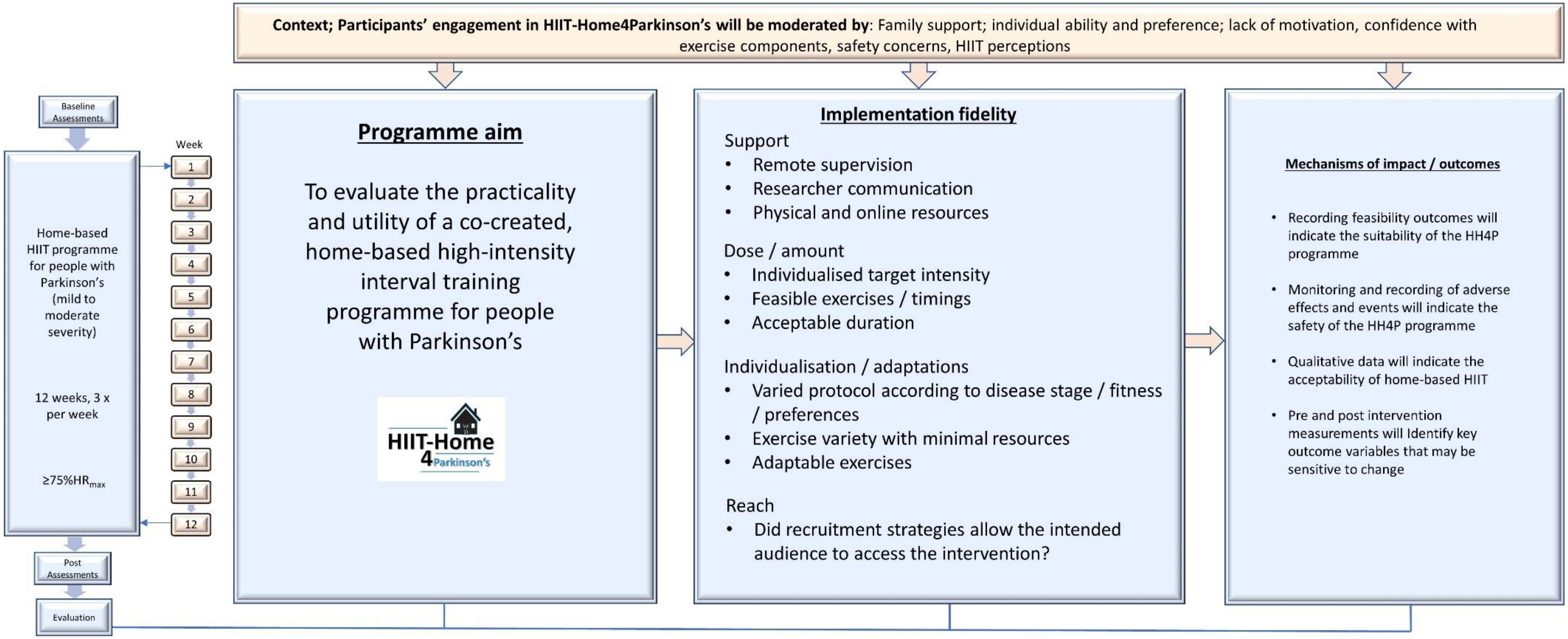

Abbreviations: HIIT – High-intensity interval training; HRmax – maximum heart rate; HH4P – HIIT-Home4Parkinson's

Tables S1–S7: Full HIIT testing results

Table S1: Round 1 HIIT testing results per participant

Participant	On / off	Sets	Work : rest ratio (s)	Number of bouts completed	Mean HR (BPM) / % HR max (work phase)	Mean RPE (work phase)	Adverse effects / events	Researcher observations
1	None	Set 1. Star Jumps	45 : 15	3	114.9 / 84.5%	13.0	None	-
		Set 2. Front arm raises (resistance bands)	45 : 15	3	111.3 / 81.9%	12.3	None	-
		Set 3.Chair sit to stand	45 : 15	3	117.5 / 86.4%	14.0	None	-
		Set 4. Lateral arm raises with weights	45 : 15	3	118.0 / 86.8%	11.3	Shoulder pain	-
		Overall	-	12 / 12	115.4 / 84.9%	12.7	Shoulder pain in set 4	Maintained form throughout. Completed exercises with relevant amplitude / cadence
2	On	Set 1. Running on the spot	45 : 15	3	127.3 / 88.4%	13.7	None	-
		Set 2. Front arm raises (resistance band)	45 : 15	3	108.2 / 75.1%	15.0	None	Amplitude of movement reduced
		Set 3. Chair sit to stand	45 : 15	3	107.5 / 74.7%	16.3	None	-
		Set 4. Lateral arm raises with weights	45 : 15	3	110.7 / 76.9%	14.3	None	-
		Overall	-	12 / 12	113.4 / 78.8%	14.8	None	Maintained frequency. Amplitude reduced periodically
3	On	Set 1. Running on the spot	45 : 15	3	116.0 / 69.9%	12.0	None	Could have increased effort (unsure of required intensity)
		Set 2. Boxing	45 : 15	3	126.4 / 76.1%	11.3	None	Doubled frequency
		Set 3. Body weight squats	45 : 15	3	126.9 / 76.4%	13.3	None	Doubled up on movement frequency to increase effort
		Set 4. Boxing	45 : 15	3	135.2 / 81.4%	13.3	None	-
		Overall	-	12 / 12	126.1 / 76.0%	12.5	None	Maintained amplitude and form. Increased effort by periodically doubling up on frequency
4	On	Set 1. Running on the spot	45 : 15	3	141.3 / 86.7%	14.3	Calf cramp in running	
		Set 2. Boxing	45 : 15	3	149.0 / 91.4%	15.3	None	Bounced to increase intensity
		Set 3. Body weight squats	45 : 15	3	148.7 / 91.2%	18.7	Knee ache in squat	-
		Set 4. Lateral arm raises with weights	45 : 15	3	140.3 / 86.1%	16.3	None	-
		Overall	-	12 / 12	144.8 / 88.8%	16.2	Calf cramp / knee ache	Squats "hard" on knees. Bounced during boxing which was observed to increase intensity
5	On	Set 1. Star jumps	45 : 15	3	136.3 / 87.4%*	18.0	None	-
		Set 2. Shoulder press with weights	45 : 15	3	136.7 / 87.6%*	17.0	None	-
		Set 3. Body weight squats	45 : 15	3	134.3 / 86.1%*	17.3	None	Imbalance and reduced movement amplitude
		Set 4. Lateral arm raises with weights	45 : 15	3	131.2 / 84.1%*	17.7	None	-
		Overall	-	12 / 12	134.6 / 86.3%*	17.5	None	Imbalance during squats, with reduced movement amplitude as participant tired

* Based on age adjusted formula 220 - age

Abbreviations: s – seconds; m – minutes; HRmax – maximum heart rate; RPE – rate of perceived exertion; BPM – Beats per minute;

Table S2: Round 1 HIIT testing results per exercise

Set / Exercise	Number of sets undertaken	Completion (%)	% sets achieving mean target HR	Mean % of HR_{max} achieved	Mean RPE achieved	Adverse effects and events	Researcher observations
1. Running on the spot	3	100	66.6	81.7	13.3	1 participant calf cramp	1 participant unsure of required effort
1. Star jumps	2	100	100	86.0	16	None	None
1. Skipping (no rope)	0	-	-	-	-	-	-
2. Boxing (crosses & jabs)	2	100	100	83.8	13.3	None	"Bouncing" observed to increase intensity
2. Overhead shoulder press + weights	1	100	100	87.6	17	None	None
2. Front arm raise + resistance band	2	100	100	78.5	13.7	None	1 participant reduced movement amplitude
3. Body weight squats	3	100	100	84.6	16.4	1 participant knee ache	Balance issues (H&Y 3). Knee pain. Reduced movement amplitude. Frequency doubled to increase intensity
3. Chair sit to stand	2	100	50	80.6	15.2	None	Unsure of position of chair. Support required
4. Boxing (Uppercuts & jabs)	1	100	100	81.4	13.3	None	None
4. Lateral arm raises + weights	4	100	100	83.5	14.9	1 participant shoulder pain	Shoulder pain when following shoulder exercises from set 2
4. Lateral arm raises + resistance bands	0	-	-	-	-	-	-

Abbreviations: HR – Heart rate; HR_{max} – Maximum heart rate; RPE – Rate of perceived exertion

Table S3: Round 2 HIIT testing results per participant

Participant	On / off	Sets	Work : rest ratio (s)	Number of bouts completed	Mean HR (BPM) / % HR max (work phase)	Mean RPE (work phase)	Adverse effects / events	Researcher observations
2	Off	Set 1. Running on the spot	45 : 15	3	128.4 / 79.8	13.0	None	-
		Set 2. Front arm raises (resistance bands)	45 : 15	3	131.3 / 81.6	15.7	None	-
		Set 3.Star jumps	45 : 15	2.5	138.6 / 86.1	19.3	Shoulder pain	Changed to jogging halfway through the final bout due to shoulder pain
		Set 4. Chair sit to stand	45 : 15	3	132.2 / 82.1	14.3	None	Used chair supports for leverage as well as balance. Improved from previous round due to support.
		Overall	-	11.5 / 12	132.6 / 82.4	15.6	Shoulder pain in set 3	Set 2 and set 3, consecutive shoulder raises and star jumps unsuitable for this participant. Increased work rate from previous round
3	On	Set 1. Running on the spot	45 : 15	3	104.1 / 62.7	12.0	None	-
		Set 2. Boxing	45 : 15	3	109.7 / 66.1	14.3	None	-
		Set 3. Star jumps	45 : 15	3	110.5 / 66.6	14.7	None	-
		Set 4. Bodyweight squats	45 : 15	3	126.0 / 75.9	15.7	None	Overbalanced forward during squats
		Overall	-	12 /12	112.6 / 67.8	14.2	None	Postponed for 90 minutes to wait for the "on" phase. Difficulty in maintaining rhythm in sets 1 and 2. Overbalancing forward during squats (on tip toes). Lunges may have been more suitable for this participant.
4	On	Set 1. Running on the spot	45 : 15	3	111.1 / 68.2	10.3	None	-
		Set 2. Boxing	45 : 15	3	123.9 / 76.0	16.0	None	Bounced during boxing to increase intensity
		Set 3. Skipping (no rope)	45 : 15	3	138.9 / 85.2	16.0	None	-
		Set 4. Forward lunges	45 : 15	3	123.0 / 75.5	15.3	None	Lunges suitable
		Overall	-	12 / 12	124.2 / 76.2	14.4	None	"Bounced" to increase intensity. Lunges observed to be suitable for this participant.

Abbreviations: s – seconds; m – minutes; HRmax – maximum heart rate; RPE – rate of perceived exertion; BPM – Beats per minute;

Table S4: Round 2 HIIT testing results per exercise

Set / Exercise	Number of sets undertaken	Completion (%)	% sets achieving mean target HR	Mean % of HR_{max} achieved	Mean RPE achieved	Adverse effects and events	Researcher observations
1 / 3. Running on the spot	3	100	33	70.2	11.8	-	One participant had difficulty synchronising movements to audio
1 / 3. Star jumps	2	92	50	76.4	17.0	Shoulder pain	Shoulder pain in set 3 following raises
1 / 3. Skipping (no rope)	1	100	100	85.2	16.0	-	
2. Boxing (crosses & jabs)	2	100	50	71.1	15.2	-	Bouncing increased intensity. Difficulty synchronising movements to audio
2. Overhead shoulder press + weights	-	-	-	-	-	-	-
2. Front arm raise + resistance band	1	100	100	81.6	15.7	-	-
4. Body weight squats	1	100	100	75.9	15.7	-	Overbalancing forward
4. Chair sit to stand	1	100	100	82.1	14.3	-	Balance aids used for leverage
4. Forward lunges	1	100	100	75.5	15.3	-	Suitable alternative

Abbreviations: HR – Heart rate; HR_{max} – Maximum heart rate; RPE – Rate of perceived exertion

Table S5: Round 3 HIIT testing results per participant

Participant	On / off	Sets	Work : rest ratio (s)	Number of bouts completed	Mean HR (BPM) / % HR max (work phase)	Mean RPE (work phase)	Adverse effects / events	Researcher observations
1	Off	Set 1. Star jumps	45 : 15	3	103.1 / 75.1	14.3	None	-
		Set 2. Front arm raises	45 : 15	3	109.3 / 80.1	13.7	None	-
		Set 3.Skipping (no rope)	45 : 15	3	119.2 / 87.6	13.7	None	-
		Set 4. Body weight squats	45 : 15	3	116.7 / 85.8	14.7	None	-
		Overall	-	12 / 12	112. 1 / 82.4	14.1	None	Exercises and sequence suitable. Good form maintained. Suitable exercises for environment. Carpet suitable surface, trainers worn.
4	On	Set 1. Running on the spot	45 : 15	3	127.2 / 78.2	12.0	None	-
		Set 2. Boxing	45 : 15	3	142.0 / 87.1	13.3	None	-
		Set 3. Star jumps	45 : 15	3	144.4 / 88.6	15.3	None	-
		Set 4. Body weight squats	45 : 15	3	134.6 / 82.6	16.0	Mild knee ache	Shallowed squats
		Overall	-	12 / 12	137.1 / 84.1	14.2	None	Carpet suitable surface. Performed in bear feet. Shallowed squats to accommodate knee ache. Maintained form throughout otherwise

Abbreviations: s – seconds; m – minutes; HRmax – maximum heart rate; RPE – rate of perceived exertion; BPM – Beats per minute;

Table S6: Round 3 HIIT testing results per exercise

Set / Exercise	Number of sets undertaken	Completion (%)	% sets achieving mean target HR	Mean % of HR_{max} achieved	Mean RPE achieved	Adverse effects and events	Researcher observations
1 / 3. Running on the spot	1	100	100	78.2	12.0	None	-
1 / 3. Star jumps	2	100	100	81.6	14.8	None	-
1 / 3. Skipping (no rope)	1	100	100	87.6	13.7	None	-
2. Boxing	1	100	100	87.1	13.3	None	-
2. Overhead shoulder press + weights	-	-	-	-	-	-	-
2. Front arm raises	1	100	100	80.1	13.7	None	-
4. Body weight squats	2	100	100	84.2	15.4	Mild knee ache	Pain subsided when squats were shallowed
4. Chair sit to stand	-	-	-	-	-	-	-
4. Forward lunges	-	-	-	-	-	-	-

Abbreviations: HR – Heart rate; HR_{max} – Maximum heart rate; RPE – Rate of perceived exertion

Table S7: Culminated HIIT testing results per exercise (excluding shoulder exercises that were removed from the protocol)

Set / Exercise	Number of sets	Set completion (%)	% sets (n) achieving mean target HR	Mean % of HR_{max} achieved	Mean RPE achieved	Adverse effects and events
1 / 3. Running on the spot	7	100	57 (4/7)	76.3	12.5	Calf cramp
1 / 3. Star jumps	6	98.7	83 (5/6)	81.4	15.8	Shoulder pain
1 / 3. Skipping (no rope)	2	100	100 (2/2)	86.4	14.9	None
2. Boxing	5	100	80 (4/5)	79.3	14.0	None
2. Overhead shoulder press + weights	1	100	100 (1/1)	87.6	17.0	None
2. Front arm raise + resistance band	4	100	100 (4/4)	79.7	14.2	None
4. Body weight squats	6	100	100 (6/6)	83.0	16.0	Knee ache
4. Chair sit to stand	3	100	67 (2/3)	81.1	14.9	None
4. Forward lunges	1	100	100 (1/1)	75.5	15.3	None

Abbreviations: HR – Heart rate; HR_{max} – Maximum heart rate; RPE – Rate of perceived exertion

Tables S8–S16: Examples of focus group transcript data

Table S8: PPI focus group 1 relating to "Concerns" sub-themes

Main theme	Sub-theme	Examples of focus group transcript data
**HIIT exercise concerns**	Time restraints	"You've still got to live and have got lives…it's trying to fit it all in…"
	Individual capacity	"I think it would depend on the individual, concerning where you are at when you start…"
	On / off periods	"You also have the issue of your on / off periods, timing it to fit it in…"
	Space restraints	"The point is where do you do it…if you've got a small home?"
	Coordination	"When you try and do legs and arms, I know other people have found it difficult as well, trying to coordinate both of those things at the same time."
**Programme delivery concerns**	Supervision / injury	"My thought would be am I doing it properly…because you are not being supervised?"
	Motivation	"Yes…it's motivation with me. It's too easy for other things to get in the way."
	No social element	"In an ideal world, I'd like to be able to go somewhere so that it becomes a social thing…"
	Screening	"So would you screen for comorbidities?"

Table S9: PPI focus group 1 relating to “Opportunities / motivators” sub-themes

Main theme	Sub-theme	Examples of focus group transcript data
**HIIT exercise opportunities / motivators**	Mode	“I’m part of a group that’s just been set up for boxing based on coordination, muscle memory and exercise… …everyone in the group is really enjoying it, finding it useful.”
	Intervals	“It seems to be similar intervals to what we’ve been doing which seems to work.”
	Adaptations	“We could look at progressing the time (of work periods) maybe more so than the intensity…”
	Equipment	“Resistance bands…” (Would be good to include), “Handheld weights”
**Programme delivery opportunities / motivators**	Home-based convenience	“If you can do it in your home, you are perhaps more likely to find that little block of half an hour and get on with it and do it.”
	Remote support	“It’s useful to have some online guidance. I thought a video showing good form and a video showing bad form.”
	Evidence of change as a motivator	“Yeah, if you see good enough change…that would be your motivator.”
	Duration / frequency	“I think the 12 weeks is quite good because then all of us can then try to devote the next 12 weeks as much as possible.”
	Monitoring equipment	“I’ve got a fit bit…it’s got a heart monitor on it…”

Table S10: Clinician focus group 1 relating to “Barriers and considerations” sub-themes

Main theme	Sub-theme	Examples of focus group transcript data
**HIIT exercise barriers and considerations**	Parkinson’s severity differentiation	“You have to think about what you are providing for 1’s and 2’s, is not what you are providing for 3’s and 4’s on the Hoehn and Yahr scale.”
	Reduced movement amplitude	“Because there is that cognitive issue of trying to keep each movement the same size when you are repeating movements – the first one is big, the second is okay and the third is a bit smaller…”
**Programme delivery concerns**	Remote support	“Few people have got a DVD player”, “Paper copies of the exercises - it’s not enthusing people.”
	Licensing	“Just thinking about music licensing…it can be tricky.” “There’s a lot of rules and regulations.”
	Motivation	“It’s motivation then, the social side, that’s a bit more tricky.”
	Outcome measures (PDQ)	“Almost all of the (exercise) interventions were not long enough to make a difference to the PDQ.”
	Physio costs	“The big cost is always the physio time…”

Table S11: Clinician focus group 1 relating to "Opportunities and facilitators" sub-themes

Main theme	Sub-theme	Examples of focus group transcript data
**HIIT exercise opportunities and facilitators**	Rhythmic cueing	"I think if it was cued to a beat of some kind you'd get a bigger volume (amplitude) of movement." "Have the music for the appropriate number of seconds, and the right beat…"
	Differentiation	"It might be the prep (aration) that's different, the lead in might be much longer before you get to the high intensity." "You could have a choice of similar. exercises…"
	Modality	"Core stability and posture (in the warm-up) would be the focus for me…" "I could understand the boxing…and you could add the coordination…"
**Programme delivery opportunities and motivators**	Remote support	"If you have the initial training, maybe say that every couple of weeks there is a check-in with a physiotherapist?" "People can download the exercises they need and have a folder on their tablet…"
	Outcome measures; change / motivation	"Your outcome measures are going to need to include something that is meaningful to them (service users), and that they can measure really easily."
	Training	I would front load it…and then I think your supervision could drop right off. I would suggest once they've got it they've got it."
	Social motivation	"But as they start as a group, could they maybe check-in as a group?"

Table S12: PPI focus group 2 relating to "outcomes" and "randomisation"

Main theme	Sub-theme	Examples of focus group transcript data
**Outcome measures – acceptability and importance**	Brain-derived neurotrophic factor acceptable and important	"No problem with that, that's acceptable, I'm used to it…" (Blood tests)
	VO_{2max} acceptable procedure	"It was fine the first time, no problem with that…"
	Physical activity, 2 weeks accelerometery acceptable	"I'd certainly wear one (accelerometer). I'd be quite happy wearing something on the waist band."
	30 second sit to stand acceptable but not suitable as fortnightly "motivator"	"The fitness thing, it's good to see it's making a difference…but what we really want to know is it slowing the progression of Parkinson's?
	Rate of perceived exertion concern with judgement	"That was the one I had concerns that I was giving the right information…"
	Unified Parkinson's Disease Rating Scale part III important and acceptable	"That would be a good idea. You have to show that it's (exercise) is having a benefit over and above making you fitter."
	Adherence, completion, adverse effects and events self-report diary acceptable	"No problem, as long as it's fairly simple stuff…"
	Ox-PAQ important and acceptable	"I think it's useful, form the point of view of monitoring where we are with Parkinson's."
**Randomisation**	Unacceptable for 1/3 participants	"Disappointed to be honest, I'd want to be on the exercise side I'm afraid."

Table S13: PPI focus group 2 relating to "HIIT protocol"

Main theme	Sub-theme	Examples of focus group transcript data
**Specific points regarding the undertaken HIIT protocol**	General thoughts, challenging and enjoyable	"I enjoyed it. It was quite tough at the end, but yeah it was good."
	Alternative exercises – core exercises, leg raises	"Maybe include something that works the core muscles?"
	Accompaniment acceptable, but requires variation	"It (differing musical styles) would also suit our moods, to encourage us…"
	Pre-recorded verbal encouragement acceptable	"I don't have a problem with that. It keeps you motivated."
	Visible heart rate monitor acceptable and useful	"When you get used to the training you would be able to feel that you are working at the right intensity, but maybe until that point?"
	Squats & sit to stand require support / fixture and fittings damage potential	"If you are repeatedly doing it (sit to stand) the chair works backwards…"<br>"Putting the chair against the wall, am I going to mark it?"

Table S14: Clinician focus group 2 relating to "outcomes" and "programme delivery"

Main theme	Sub-theme	Examples of focus group transcript data
**Outcome measures**	Fortnightly motivational check- in	"I think these motivational chats, when you do contact and motivate it's really lovely…"
	Range of outcomes to allow for individualised motivation	"Maybe for some fitness is the goal, for others it might be about how many stairs they can go up…"
	Additional motivational check-ins resource intensive	"These (check-ins) will add to researcher time…"
	Goal Attainment Scale achievable procedure but problematic	"The GAS (Goal Attainment Scale) is difficult to standardise…"
**Programme delivery**	Proposed programme has multiple options	"It sounds comprehensive. It's not one size fits all, so you've got options there for people."
	Ensure choice of options to avoid overloading	"Make it clear that they don't have to do all of them at once."
	Use of Smart phone application	"Participants could use their phone, that would be ideal…"

Table S15: Clinician focus group 2 relating to “HIIT protocol”

Main theme	Sub-theme	Examples of focus group transcript data
**Specific points regarding the HIIT protocol**	Squats and sit-to-stand balance safety and alternatives	“Maybe go for single leg lunges, so you’ve got that one flat foot?”<br>“You could hold on to a chair, or near a wall…”
	Core exercises stimulate inadequate intensity	“I’m not sure you’d get your heart rate up doing that…” (Core exercises)
	Shoulder exercises overly similar	“It’s not so much about the shoulder exercises per se, it’s because they are in the same direction…”
	Alternative exercises to facilitate clinical prescription	“As a therapist you could say pick the one you are happy with…”
	Exercise sequence alternatives	I think the upper body, lower body (sequence) is fine, just mix it up a bit.”<br>You could do cardio, something different, cardio, something different…”

Table S16: PPI focus group 3 relating to "Increase engagement" and "Reduce engagement" in the HH4P developed exercise protocol

Main theme	Sub-theme	Examples of focus group transcript data
**Increase engagement**	Additional opportunities within initial home visit: Environment / required space	"I think that's a really key thing to be worked out in that initial home visit. Where is this going to happen? What have you got on the floor? What's surrounding you?"
	Additional opportunities within initial home visit: Engagement with partner	"Going into people's homes…explain what they should be doing and the benefits to the partner…"
	Partner support	"I'd want to know what she (exercise participant) was doing and how she should be doing it."
	Potential to exercise outside	"If it was a nice day I would do the exercise outside…"
**Reduce engagement**	Exercise environment: Surface	I just feel, if your feet stick to it (carpet), you can't swivel of move so freely on it…"
	Exercise environment: Space	"You haven't got quite the width of the room. I'd be worried about running into the cupboards!"
	Exercise environment: Damage	"When I wear a hole in the carpet I'll be looking for contributions!"
	Lack of partner support	"They (partners) might turn around and say it's nothing to do with me…"
	Motivation	"It depends on your attitude. I mean I'm all fight, but a lot of people aren't…"
	Footwear	"So I put trainers on and I'll be sticking to the floor to start with."

Figures S2–S6: Graphical examples of participant heart rate data (HIIT round 1)

Figure S2: Participant 1

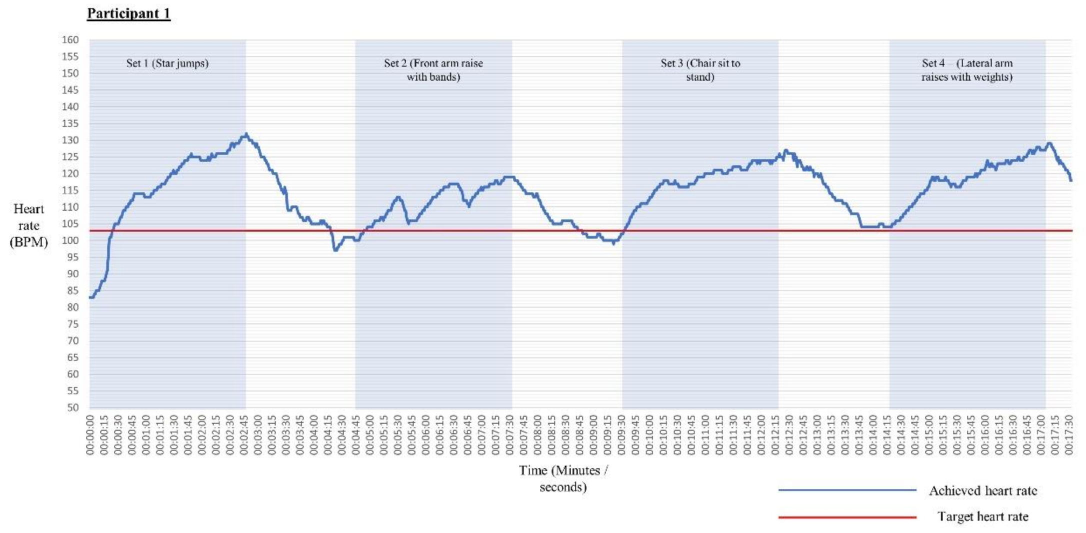

Figure S3: Participant 2

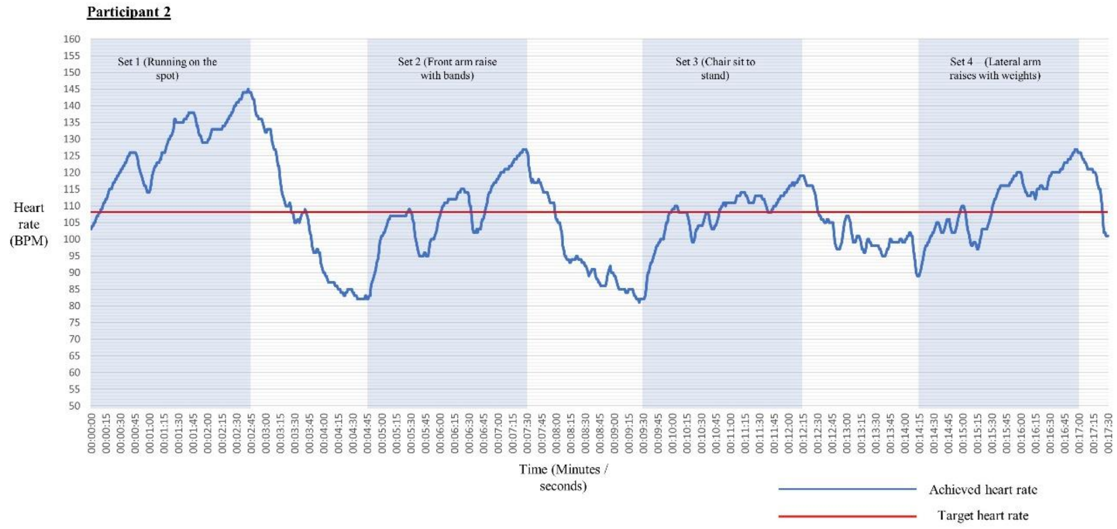

Figure S4: Participant 3

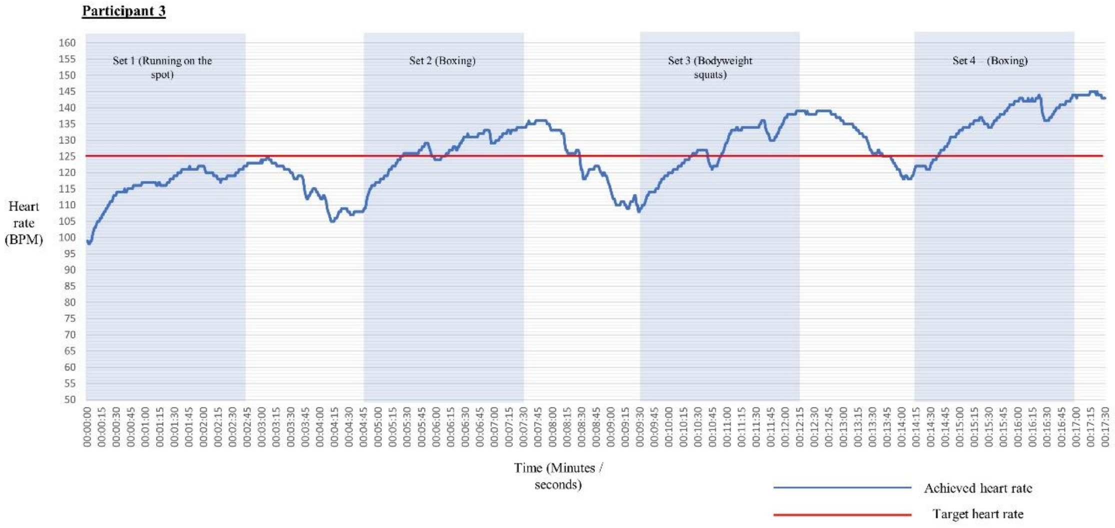

Figure S5: Participant 4

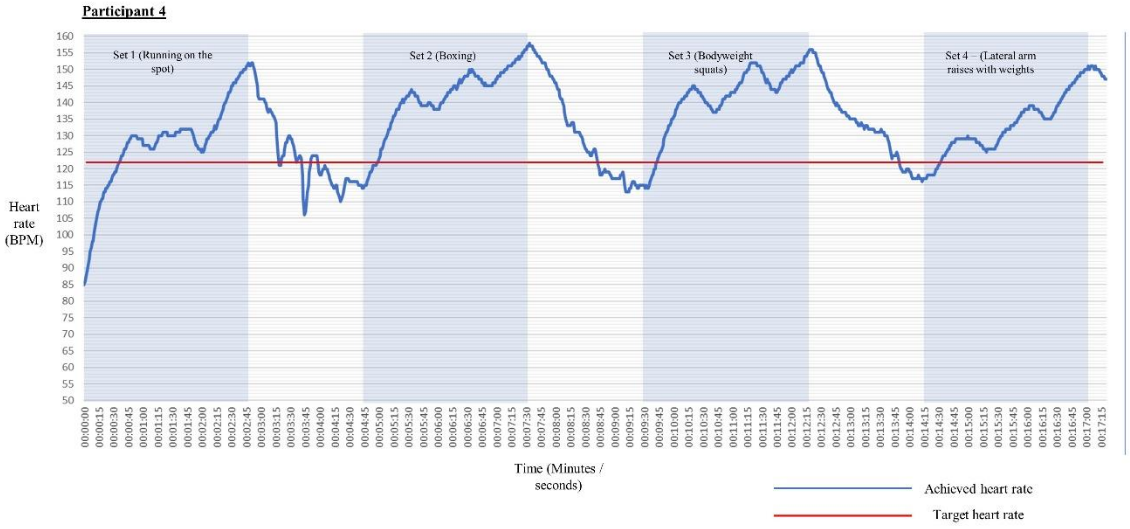

Figure S6: Participant 5

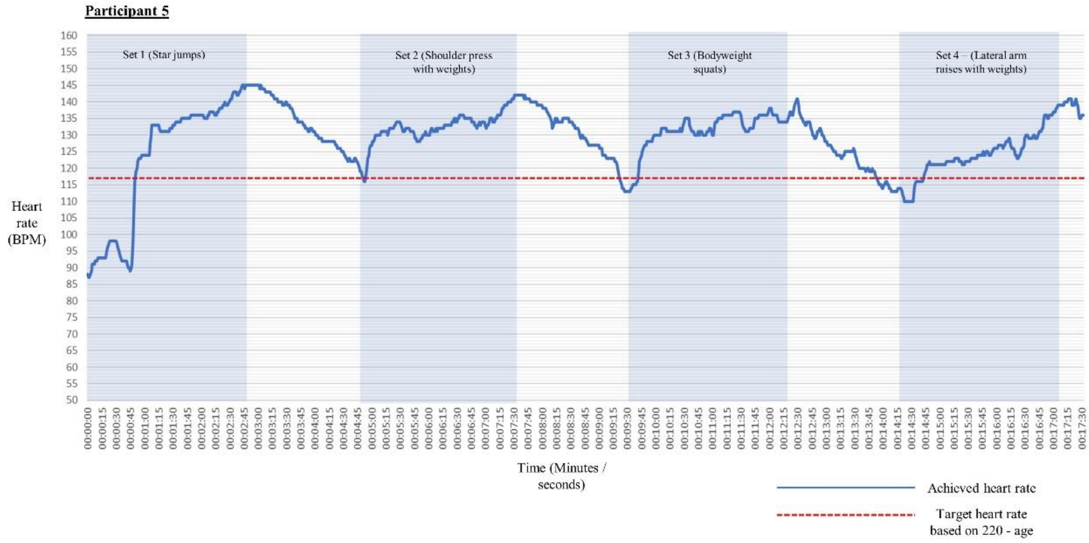

Supplement: Supplementary file 1 [file ijerph-20-05671-s001.zip › ijerph-2331220-supplementary.pdf]
